# Exploring antibody–antigen binding affinity using degree-based topological indices: a graph-theoretical approach

**DOI:** 10.3389/fchem.2026.1899937

**Published:** 2026-08-27

**Authors:** Flemin Sajeev, Roy S

**Affiliations:** School of Advanced Sciences, Vellore Institute of Technology, Vellore, India

**Keywords:** antibody-antigen interaction, binding affinity, edge-partitioning method, molecular graph, topological indices

## Abstract

The binding affinity between an antibody and an antigen, measured by the equilibrium dissociation constant $\left(K_{d}\right)$ and the associated Gibbs free energy of binding (ΔG) , is key to immune recognition and the design of therapeutic antibodies. This study explores whether degree-based topological indices, derived from molecular graph models of fifteen antibody–antigen Fab complexes from the Protein Data Bank, contain structural information related to binding affinity. Fifteen indices were calculated using an edge-partitioning technique and analyzed against two thermodynamically based response variables, $pK_{d}$ and experimental ΔG , employing linear, quadratic, and cubic models, while keeping the untransformed $K_{d}$ as an additional point of reference. Cubic models reached statistical significance against both $pK_{d}$ and ΔG independently, while linear and quadratic models did not reach significance for either response variable. Additionally, a size benchmark comparing total atom count and edge count against all three response variables reproduced the performance of the best performing topological index at every model order, confirming that the observed associations reflect molecular size rather than connectivity-specific structural information. The results demonstrate that degree-based topological indices primarily reflect overall molecular size rather than connectivity-specific structural information. This work establishes a solid foundation for developing more focused hybrid descriptor methods for analyzing antibody–antigen affinity.

## 1 Introduction

Antibody-antigen interactions are fundamental to immune recognition and play a crucial role in therapeutic antibody design, vaccine development, and diagnostics (Huang et al., 2020; Kapingidza et al., 2020; Kumagai and Tsumoto, 2001; Nicholson, 2016; Webster et al., 1994). The strength of this interaction is commonly measured using the equilibrium dissociation constant $\left(K_{d}\right)$ , which reflects how strongly an antibody binds to its antigen, and the associated Gibbs free energy of binding (ΔG) , which is thermodynamically related to $K_{d}$ on a logarithmic scale (Li and Zhu, 2010; Lu et al., 2020; Norman et al., 2020). Therefore, accurate estimation of binding affinity is crucial for comprehending molecular interactions and creating potent therapeutic medicines (Bi et al., 2023; Guest et al., 2021; Mikolajek et al., 2022; Rhoden et al., 2016; Yang et al., 2023a; Yang et al., 2023b). Even though they are dependable, experimental techniques including surface plasmon resonance (SPR), enzyme-linked immunosorbent assays (ELISA), and isothermal titration calorimetry (ITC) are frequently costly, time-consuming, and unsuitable for large-scale study (Ma et al., 2018; Pathare et al., 2014).

To overcome these limitations, computational methods for calculating binding affinity from structural information have been widely investigated. Deep learning methods have progressed quickly in recent years. Wang et al. introduced AntiFormer, a graph-based large language model that incorporates sequence information in a graph framework to predict antibody-antigen binding affinity. It shows strong performance across several benchmark datasets (Wang et al., 2024). Related sequence-based methods include AttABseq, an attention-based model that predicts binding affinity changes from protein sequences (Jin et al., 2024), and MVSF-AB, a multi-view learning framework that combines residue-level and semantic sequence features (Li et al., 2025). Chen Liu et al. presented ProtAttBA, a sequence-based approach for predicting binding affinity changes using attention mechanisms (Liu et al., 2025). Structure-based graph neural network methods have also been created for the related problem of predicting protein-protein binding affinity. Wang et al. proposed a topology-based network tree that uses persistent homology for protein-protein complexes. This embeds structural information into topological invariants for further machine learning (Wang et al., 2020). ProAffinity-GNN combines a curated structural dataset with graph neural networks to estimate protein-protein affinity (Liu, 2024). Despite the strong predictive performance of these models, they require a lot of computing power. They often act as black boxes, providing limited structural insight, especially in situations with scarce data. These models usually rely on large training datasets and complex learned feature representations.

In contrast, descriptor-based approaches like Quantitative Structure-Activity Relationship (QSAR) provide a simpler and more interpretable approach for this study. Chemical graph theory offers an effective means of representing molecular structures as molecular graphs, with atom bonds represented as edges and atoms as vertices (Bondy and Murty, 1979; Trinajstić, 2018). These representations can be used to calculate topological indices, which are numerical descriptors that describe the structural properties of molecules. When considering different chemical structures, topological indices have been widely used in QSAR and QSPR investigations (Gutman, 2013; Rouvray, 1987). Among these, degree-based topological indices are easy to calculate and useful for characterizing molecular graph connectivity patterns (Hosamani et al., 2017; Katritzky et al., 1995; Keyvanpour and Shirzad, 2021; Kiralj and Ferreira, 2009). However, their application to complex biomolecular systems such as protein–protein and antibody–antigen interactions remains relatively limited, which presents an opportunity for further exploration (Arockiaraj et al., 2024a; Dearden, 2017; Gayathri and Roy, 2025a; Gayathri and Roy, 2025b; Gnanaraj et al., 2023; Jyothish and Roy, 2024; Jyothish and Roy, 2025; Jyothish and Santiago, 2025; Kiralj and Ferreira, 2009; Randić, 1996; Roy, 2017).

This work presents an exploratory investigation into whether degree-based topological indices, derived from covalent heavy-atom graph representations of antibody–antigen Fab complexes obtained from the Protein Data Bank, carry structural information associated with binding affinity. A set of fifteen degree-based indices is computed using an edge partitioning method and evaluated against two thermodynamically grounded measures of binding affinity, $pK_{d}=-\log_{10}\left(K_{d}\right)$ and experimental ΔG , using linear, quadratic, and cubic regression models, with untransformed $K_{d}$ retained as a secondary point of comparison. To assess whether the observed associations reflect genuine structural signal or are driven by overall molecular size, a benchmark comparison against total atom and edge count is additionally performed. The objective of this study is to build an empirical foundation for more focused graph-theoretical methods to antibody–antigen affinity modeling in subsequent work by methodically analyzing what these descriptors capture and what they do not, when applied to large biomolecular complexes.

## 2 Materials and methods

### 2.1 Antigen-antibody complex

**FIGURE 1:**
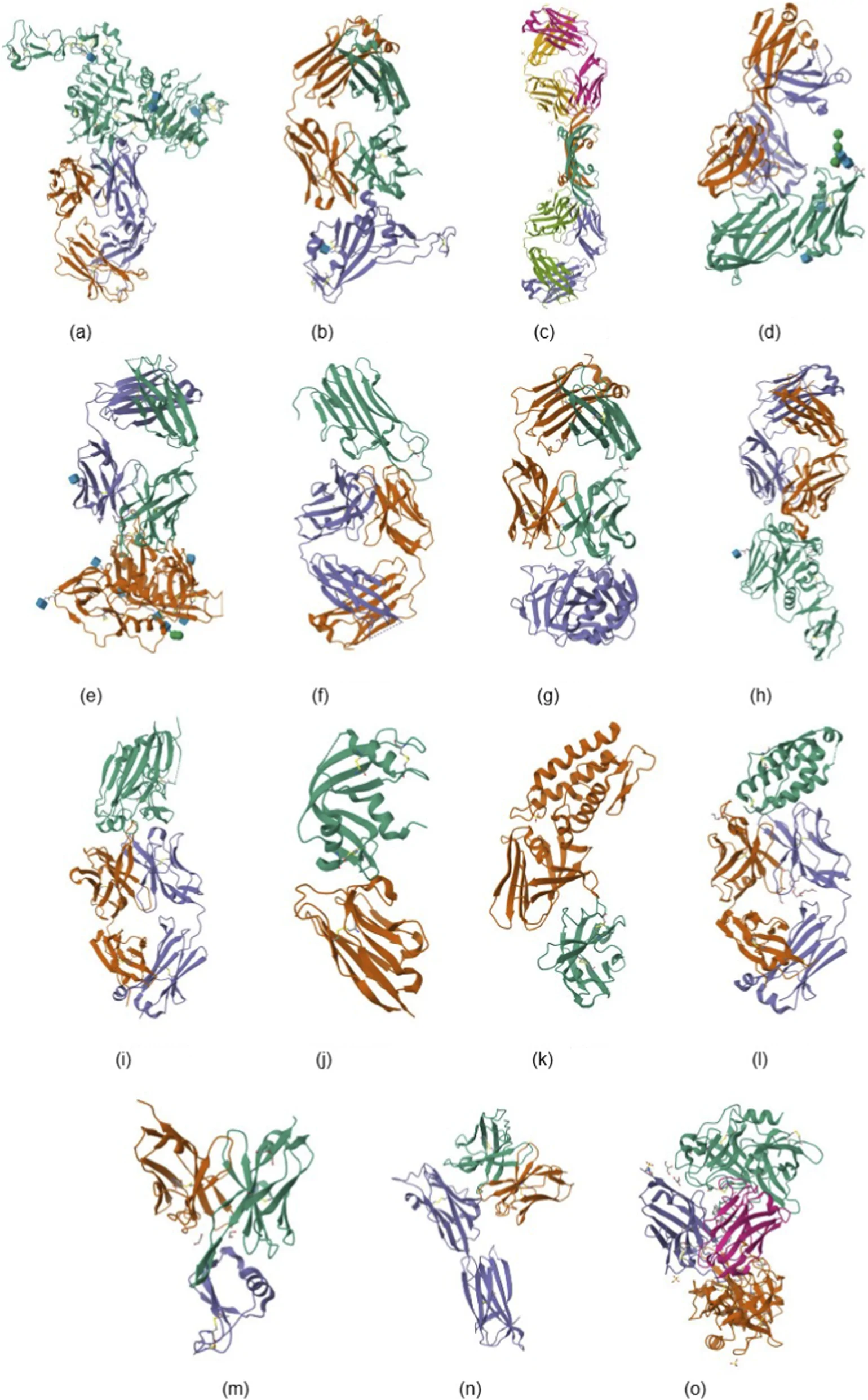
Structures of antibody-antigen fab complex. **(a)** 1S78, **(b)** 2DD8, **(c)** 2FJG, **(d)** 3MJ9, **(e)** 3U7Y, **(f)** 3WD5, **(g)** 4ETQ, **(h)** 4FP8, **(i)** 4M5Z, **(j)** 4POU, **(k)** 4Y7M, **(l)** 5C7X, **(m)** 5CBA, **(n)** 5GRJ, **(o)** 5HGG.

The Antibody-Antigen Fab structures used in this article were obtained from the Protein Data Bank (PDB) (https://www.rcsb.org/) (Kapingidza et al., 2020; Kumagai and Tsumoto, 2001). These structures were chosen based on available binding affinity values from the antibody-antigen docking and affinity prediction benchmark (https://github.com/piercelab/antibody_benchmark) (Guest et al., 2021; Yang et al., 2023a). All structures were obtained by X-ray diffraction, an experimental method used to acquire high resolution three-dimensional structures of antibody-antigen fragments. The resolutions of the selected structures range from 1.6Å to 3.5Å (Angstrom), indicating that these are high-resolution models of the chemical interactions at the binding interface. Prior to analysis, each PDB structure was preprocessed to remove water molecules using PyMOL. Several structures in the dataset contained crystallographically duplicated biological assemblies, where multiple copies of the same antibody-antigen complex were present in a single asymmetric unit due to crystal packing. For these structures, a single representative biological assembly was kept by removing the extra chain copies, ensuring that each entry in the dataset corresponds to exactly one antibody-antigen complex. Additionally, atoms recorded with alternate location indicators, which represent alternate crystallographic forms of the same atom, were excluded, keeping only the main form. The structures that needed chain deduplication were 1S78, 2FJG, 4ETQ, 4FP8, 4Y7M, 5C7X, 5CBA, and 5HGG. The structures affected by altloc were 3U7Y, 4FP8, 4POU, 4ETQ, and 5HGG. Figure 1 shows the Antibody-Antigen Fab structures used in this study.

### 2.2 Binding affinity

Binding affinity can be evaluated using the equilibrium dissociation constant $\left(K_{d}\right)$ and the change in Gibbs free energy (ΔG) (Ma et al., 2018; Pathare et al., 2014). The equilibrium dissociation constant can be defined as:

$$K_{d}=\frac{\left[Ab\right]\left[Ag\right]}{\left[Ab-Ag\right]}$$

where [Ab] and [Ag] are the concentrations of free antibody and antigen, respectively, and [Ab-Ag] is the concentration of the antibody–antigen complex. A low $K_{d}$ value indicates strong binding, while a high $K_{d}$ value corresponds to weak binding. Experimental $K_{d}$ values for the fifteen complexes, obtained from the Pierce Laboratory benchmark, are given in Table 1 and range from 0.007 nM to 500 nM, spanning nearly five orders of magnitude. Because binding free energy is thermodynamically related to $K_{d}$ through:

$$\Delta G=RT\ln K_{d}$$

where R is the universal gas constant and T is the absolute temperature. Two thermodynamically grounded response variables are therefore used as the primary measures of binding affinity in this study. The first is $pK_{d}$ , defined as:

$$pK_{d}=-\log_{10}\left(K_{d}\right)$$

where $K_{d}$ is expressed in molar units. Since $pK_{d}$ is proportional to −ΔG , regression against $pK_{d}$ is thermodynamically more appropriate than regression against untransformed $K_{d}$ . The second primary response variable is the experimental Gibbs free energy of binding ( ΔG , kcal/mol), obtained directly from the Pierce Laboratory benchmark for all fifteen complexes. Untransformed $K_{d}$ is retained as a secondary response variable for comparative purposes, following the convention used in prior antibody-antigen affinity benchmark studies (Guest et al., 2021; Rhoden et al., 2016).

**TABLE 1:** Binding affinity values for the fifteen antibody-antigen complexes.

Structure	$K_{d}$ (nM)	$pK_{d}$	ΔG (kcal/mol)
1S78	500	6.301	− 8.45
2DD8	20	7.699	− 10.50
2FJG	20	7.699	− 10.50
3MJ9	8	8.097	− 11.05
3U7Y	160	6.796	− 9.27
4FP8	430	6.367	− 8.83
4M5Z	10	8.000	− 11.10
4POU	157	6.804	− 9.28
3WD5	0.115	9.939	− 13.56
4ETQ	0.18	9.745	− 13.29
4Y7M	1.61	8.793	− 11.79
5C7X	0.007	11.155	− 15.22
5CBA	0.3715	9.430	− 13.38
5GRJ	0.0421	10.376	− 14.15
5HGG	0.054	10.268	− 14.01

### 2.3 Molecular graph construction

The molecular graph G of each antibody–antigen complex was constructed as a covalent heavy-atom graph. Atom coordinates were extracted only from ATOM records, while HETATM records were excluded. Custom MATLAB routines parsed ATOM records directly from the PDB files. An edge was defined between any two atoms if their pairwise Euclidean distance was within a threshold of 1.9 (Gayathri et al., 2026). This is consistent with the bond-length range of covalent bonds between heavy atoms in biological macromolecules. This threshold captures covalent connectivity and excludes non-bonded van der Waals contacts, which start at about 2.7–2.8 Å (Bondi, 1964; Rappé et al., 1992). Hydrogen atoms are not included as vertices in the graph since they are mostly unresolved at the crystallographic resolutions in this dataset. Graph construction followed the procedure reported by Gayathri et al. (Gayathri et al., 2026), implemented in MATLAB.

### 2.4 Edge-partitioning method

Let G be the molecular graph of an antibody–antigen complex, with vertex set V(G) and edge set E(G) . For each vertex v∈V(G) , the degree $d_{v}$ is defined as the number of edges incident to v . The edge-partitioning method assigns each edge e=(u,v)∈E(G) to a partition class determined by the unordered pair of endpoint degrees $\left(d_{u},d_{v}\right)$ , with the convention $d_{u}\leq d_{v}$ . All edges sharing the same degree pair belong to the same partition class. This representation provides a compact summary of the graph’s connectivity structure. The edge partition was computed for all fifteen preprocessed structures using MATLAB, and the resulting partition tables are given in Section 3.1.

### 2.5 Degree-based topological indices

Topological indices are the numerical descriptors derived from the molecular graph that capture the structural information which can be used to describe its chemical and physical properties. The Wiener index, which was introduced in the late 1940s and showed a strong correlation between molecular branching and hydrocarbon boiling points, was one of the early attempts to correlate molecular structure with physicochemical properties that gave rise to the concept of topological indices. Since then, numerous topological indices have been created to represent various facets of molecular topology. Among these, degree-based topological indices have garnered a lot of interest because of their ease of use, high predictive power, and computational efficiency (Ali et al., 2014; Estrada and Uriarte, 2001; Furtula et al., 2014; Furtula and Gutman, 2015; Gutman, 2013; Gutman et al., 2014; Gutman et al., 2018; Shirdel et al., 2013; Zhong, 2012). In this paper, we consider 15 degree-based topological indices, the mathematical expressions are given in Table 2.

**TABLE 2:** Degree-based topological indices.

Topological index	Mathematical expression
First zagreb	$M_{1}\left(G\right)=\sum_{uv\in E\left(G\right)}\left(d_{u}+d_{v}\right)$
Second zagreb	$M_{2}\left(G\right)=\sum_{uv\in E\left(G\right)}\left(d_{u}d_{v}\right)$
Reduced second zagreb	$RM_{2}\left(G\right)=\sum_{uv\in E\left(G\right)}\left(d_{u}-1\right)\left(d_{v}-1\right)$
Randić	$R\left(G\right)=\sum_{uv\in E\left(G\right)}\frac{1}{\sqrt{d_{u}d_{v}}}$
Reciprocal randić	$RR\left(G\right)=\sum_{uv\in E\left(G\right)}\sqrt{d_{u}d_{v}}$
Hyper zagreb	$HM\left(G\right)=\sum_{uv\in E\left(G\right)}{\left(d_{u}+d_{v}\right)}^{2}$
Augmented zagreb	$AZ\left(G\right)=\sum_{uv\in E\left(G\right)}{\left(\frac{d_{u}d_{v}}{d_{u}+d_{v}-2}\right)}^{3}$
Harmonic	$H\left(G\right)=\sum_{uv\in E\left(G\right)}\frac{2}{d_{u}+d_{v}}$
Forgotten	$F\left(G\right)=\sum_{uv\in E\left(G\right)}\left(d_{u}^{2}+d_{v}^{2}\right)$
Symmetric division	$SD\left(G\right)=\sum_{uv\in E\left(G\right)}\frac{d_{u}^{2}+d_{v}^{2}}{d_{u}d_{v}}$
Atom bond connectivity	$ABC\left(G\right)=\sum_{uv\in E\left(G\right)}\sqrt{\frac{d_{u}+d_{v}-2}{d_{u}d_{v}}}$
Geometric arithmetic	$GA\left(G\right)=\sum_{uv\in E\left(G\right)}\frac{2\sqrt{d_{u}d_{v}}}{d_{u}+d_{v}}$
Inverse sum	$IS\left(G\right)=\sum_{uv\in E\left(G\right)}\frac{d_{u}d_{v}}{d_{u}+d_{v}}$
Sum connectivity	$SC\left(G\right)=\sum_{uv\in E\left(G\right)}\frac{1}{\sqrt{d_{u}+d_{v}}}$
Sombor	$So\left(G\right)=\sum_{uv\in E\left(G\right)}\sqrt{d{\left(u\right)}^{2}+d{\left(v\right)}^{2}}$

### 2.6 Size benchmark variables

To assess whether the correlations between topological indices and binding affinity reflect structural information specific to molecular connectivity or are attributable to overall molecular size, two size proxy variables were computed for each complex from the preprocessed PDB structures. The total atom count N is the number of vertices in the molecular graph G , equal to the number of ATOM records retained after preprocessing. The total edge count E is the number of edges in G , determined by the 1.9 Å covalent bond threshold described in Section 2.3. Both N and E scale directly with molecular size and carry no information about the distribution or pattern of connectivity within the graph. The values of N and E for all fifteen structures are reported in Table 3. Regression of N and E against $K_{d}$ , $pK_{d}$ , and ΔG using the same polynomial models applied to the topological indices provides a direct empirical test of whether the indices contribute information beyond molecular size alone.

**TABLE 3:** Number of atoms and edges in the antibody–antigen complexes.

Structure	Atoms (N)	Edges (E)
1S78	7703	7883
2DD8	4740	4863
2FJG	4020	4118
3MJ9	4995	5105
3U7Y	5970	6107
4FP8	5477	5601
4M5Z	4928	5061
4POU	1826	1860
3WD5	4432	4532
4ETQ	5133	5265
4Y7M	2815	2883
5C7X	4062	4160
5CBA	2328	2379
5GRJ	3398	3465
5HGG	2894	2958

### 2.7 Performance metrics and regression models

The performance of the QSAR models that correlate degree-based topological indices and antibody-antigen binding affinity was evaluated using four statistical models: Regression coefficient, coefficient of determination, root mean square error and p -value. These statistical models allow us to quantify relationships, estimate predictions, and analyze the effect of predictors on the output (Arockiaraj et al., 2023; Arockiaraj et al., 2024b; Kiralj and Ferreira, 2009; Zaman et al., 2024; Havare, 2019).

Regression coefficient (R):

Regression coefficient measures the linear relationship between the predicted and experimental binding affinity values. Its range is −1 to 1. The value 0.7–0.9 indicates strong correlation.

Coefficient of determination ( $R^{2}$ ):

Coefficient of determination is a statistical metric used in regression analysis that measures how well the model explains the variability in the data and helps in comparing different regression models and selecting the best model in the prediction. Its value ranges from 0 to 1. The values that are close to 1 indicate high explanatory power.

Root Mean Square Error (RMSE):

RMSE calculates the average magnitude of the predicted errors that is difference between the measured value and the predicted value. It is always non negative and the lower RMSE indicates better predictive accuracy. It is given by:

$$\text{RMSE}=\sqrt{\frac{1}{n}\overset{n}{\underset{i=1}{\sum }}{\left(y_{i}-{\hat{y}}_{i}\right)}^{2}}$$

where $y_{i}$ is the observed value and ${\hat{y}}_{i}$ is the predicted value.

p - value:

The p -value or the probability value measures the model’s significance. When the p -value ≤0.05 it indicate that the predictor is statistically significant.

Regression models:

The association between each descriptor topological index or size proxy and binding affinity was examined using linear, quadratic, and cubic polynomial regression models:

$$\text{Linear:}\;y=\alpha_{1}x+\alpha_{2},$$

$$\text{Quadratic:}\;y=\alpha_{3}x^{2}+\alpha_{4}x+\alpha_{5},$$

$$\text{Cubic:}\;y=\alpha_{6}x^{3}+\alpha_{7}x^{2}+\alpha_{8}x+\alpha_{9}.$$

where y denotes the response variable ( $pK_{d}$ , ΔG , or $K_{d}$ ), x denotes the predictor variable, and $\alpha_{i}$ (i=1,…,9) are fitted constants. All predictor variables were mean-centred and scaled to unit standard deviation prior to fitting, ensuring numerical stability in polynomial regression. All models were fitted using MATLAB.

**FIGURE 2:**
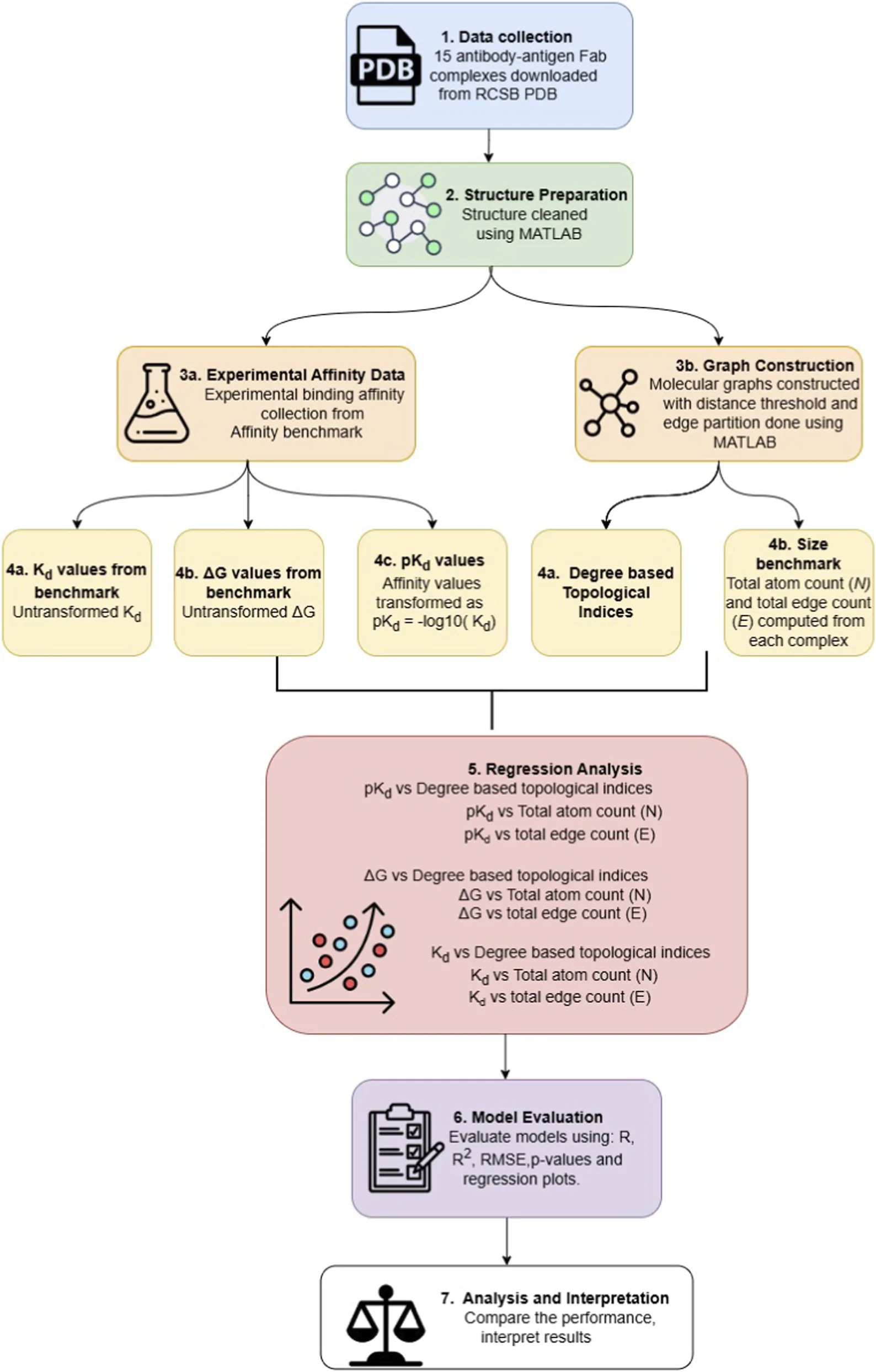
Methodological overview.

Figure 2 depicts the study’s methodology flow chart.

## 3 Results

### 3.1 Edge partition of antibody-antigen complexes

Molecular graphs of the fifteen antibody-antigen Fab complexes were created as per the procedure in Section 2.3, and the edge partition of each graph was calculated as per the procedure explained in Section 2.4. The edge partitions of all fifteen structures can be seen in Table 4. Across all fifteen structures, the total number of edges ranges from 1860 (4POU) to 7883 (1S78).

**TABLE 4:** Edge partition based on degree pairs $\left(d_{u},d_{v}\right)$ for the 15 antibody–antigen structures.

Structure	(1, 2)	(1, 3)	(2, 2)	(2, 3)	(3, 3)
1S78	222	2077	911	3440	1233
2DD8	153	1250	570	2083	807
2FJG	143	1044	491	1775	665
3MJ9	160	1348	589	2180	828
3U7Y	203	1572	733	2623	976
4FP8	185	1459	640	2417	900
4M5Z	167	1274	601	2191	828
4POU	61	486	227	813	273
3WD5	135	1204	503	1967	723
4ETQ	182	1329	596	2320	838
4Y7M	66	777	333	1254	453
5C7X	134	1072	499	1776	679
5CBA	86	609	282	1022	380
5GRJ	107	933	381	1499	545
5HGG	97	755	366	1295	445

### 3.2 Computed topological index values

The 15 molecular graphs of antibody-antigen fragments were analyzed using the edge partitioning approach described in Table 4. Later on, degree-based topological indices are calculated. The following result describes how degree-based topological indices are found. The following result gives a sample calculation of the degree-based topological indices for the complex 1S78, derived directly from its edge partition, to illustrate how the index values in Section 3.2 were obtained.

Result 1 *Let* G *denote the molecular graph of the antibody–antigen fragment, PDB ID: 1S78. Then,*

$$\begin{array}{ll} & M_{1}\left(G\right)=37216,\;M_{2}\left(G\right)=42056,\;RM_{2}\left(G\right)=12723,\\ & R\left(G\right)\approx 3627.01,\;RR\left(G\right)\approx 17858.67,\;HM\left(G\right)=180194,\\ & AZ\left(G\right)\approx 57638.52,\;H\left(G\right)=3429,\;F\left(G\right)=96082,\\ & SD\left(G\right)\approx 19219.67,\;ABC\left(G\right)\approx 5751.46,\;GA\left(G\right)\approx 7522.54,\\ & IS\left(G\right)\approx 8594.25,\;SC\left(G\right)\approx 3663.96,\;So\left(G\right)\approx 27275.43. \end{array}$$

Let G≅ PDBID: 1S78. The edge partition of G , derived from Table 4, is as follows:

$$\begin{array}{l} M_{1}\left(G\right)=\sum_{uv\in E\left(G\right)}\left(d_{u}+d_{v}\right)\\ =222\left(1+2\right)+2077\left(1+3\right)+911\left(2+2\right)+3440\left(2+3\right)+1233\left(3+3\right)\\ =37216 \end{array}$$

$$\begin{array}{cc} M_{2}\left(G\right) & =\sum_{uv\in E\left(G\right)}d_{u}d_{v}\\ & =222\left(1\times 2\right)+2077\left(1\times 3\right)+911\left(2\times 2\right)+3440\left(2\times 3\right)+1233\left(3\times 3\right)\\ & =42056 \end{array}$$

$$\begin{array}{cc} RM_{2}\left(G\right) & =\sum_{uv\in E\left(G\right)}\left(d_{u}-1\right)\left(d_{v}-1\right)\\ & =222\left(0\times 1\right)+2077\left(0\times 2\right)+911\left(1\times 1\right)+3440\left(1\times 2\right)+1233\left(2\times 2\right)\\ & =12723 \end{array}$$

$$\begin{array}{cc} R\left(G\right) & =\sum_{uv\in E\left(G\right)}\frac{1}{\sqrt{d_{u}d_{v}}}\\ & =222\frac{1}{\sqrt{1\times 2}}+2077\frac{1}{\sqrt{1\times 3}}+911\frac{1}{\sqrt{2\times 2}}+3440\frac{1}{\sqrt{2\times 3}}+1233\frac{1}{\sqrt{3\times 3}}\\ & \approx 3627.01 \end{array}$$

$$\begin{array}{l} RR\left(G\right)=\sum_{uv\in E\left(G\right)}\sqrt{d_{u}d_{v}}\\ =222\sqrt{1\times 2}+2077\sqrt{1\times 3}+911\sqrt{2\times 2}+3440\sqrt{2\times 3}+1233\sqrt{3\times 3}\\ \approx 17858.67 \end{array}$$

$$\begin{array}{l} HM\left(G\right)=\sum_{uv\in E\left(G\right)}{\left(d_{u}+d_{v}\right)}^{2}\\ =222{\left(1+2\right)}^{2}+2077{\left(1+3\right)}^{2}+911\left({\left(2+2\right)}^{2}\right)+3440\left({\left(2+3\right)}^{2}\right)+1233\left({\left(3+3\right)}^{2}\right)\\ =180194 \end{array}$$

$$\begin{array}{l} AZ\left(G\right)=\sum_{uv\in E\left(G\right)}{\left(\frac{d_{u}d_{v}}{d_{u}+d_{v}-2}\right)}^{3}\\ =222\times {\left(\frac{1\times 2}{1+2-2}\right)}^{3}+2077\times {\left(\frac{1\times 3}{1+3-2}\right)}^{3}+911\times {\left(\frac{2\times 2}{2+2-2}\right)}^{3}+3440\times {\left(\frac{2\times 3}{2+3-2}\right)}^{3}+1233\times {\left(\frac{3\times 3}{3+3-2}\right)}^{3}\\ \approx 57638.52 \end{array}$$

$$\begin{array}{l} H\left(G\right)=\sum_{uv\in E\left(G\right)}\frac{2}{d_{u}+d_{v}}\\ =222\times \frac{2}{1+2}+2077\times \frac{2}{1+3}+911\times \frac{2}{2+2}+3440\times \frac{2}{2+3}+1233\times \frac{2}{3+3}\\ =3429 \end{array}$$

$$\begin{array}{l} F\left(G\right)=\sum_{uv\in E\left(G\right)}\left(d_{u}^{2}+d_{v}^{2}\right)\\ =222\left(1^{2}+2^{2}\right)+2077\left(1^{2}+3^{2}\right)+911\left(2^{2}+2^{2}\right)+3440\left(2^{2}+3^{2}\right)+1233\left(3^{2}+3^{2}\right)\\ =96082 \end{array}$$

$$\begin{array}{l} SD\left(G\right)=\sum_{uv\in E\left(G\right)}\left(\frac{d_{u}^{2}+d_{v}^{2}}{d_{u}d_{v}}\right)\\ =222\times \frac{1^{2}+2^{2}}{1\times 2}+2077\times \frac{1^{2}+3^{2}}{1\times 3}+911\times \frac{2^{2}+2^{2}}{2\times 2}+3440\times \frac{2^{2}+3^{2}}{2\times 3}+1233\times \frac{3^{2}+3^{2}}{3\times 3}\\ \approx 19219.67 \end{array}$$

$$\begin{array}{l} ABC\left(G\right)=\sum_{uv\in E\left(G\right)}\sqrt{\frac{d_{u}+d_{v}-2}{d_{u}d_{v}}}\\ =222\sqrt{\frac{1+2-2}{1\times 2}}+2077\sqrt{\frac{1+3-2}{1\times 3}}+911\sqrt{\frac{2+2-2}{2\times 2}}+3440\sqrt{\frac{2+3-2}{2\times 3}}+1233\sqrt{\frac{3+3-2}{3\times 3}}\\ \approx 5751.46 \end{array}$$

$$\begin{array}{l} GA\left(G\right)=\sum_{uv\in E\left(G\right)}\frac{2\sqrt{d_{u}d_{v}}}{d_{u}+d_{v}}\\ =222\times \frac{2\sqrt{1\times 2}}{1+2}+2077\times \frac{2\sqrt{1\times 3}}{1+3}+911\times \frac{2\sqrt{2\times 2}}{2+2}+3440\times \frac{2\sqrt{2\times 3}}{2+3}+1233\times \frac{2\sqrt{3\times 3}}{3+3}\\ \approx 7522.54 \end{array}$$

$$\begin{array}{l} IS\left(G\right)=\sum_{uv\in E\left(G\right)}\frac{d_{u}d_{v}}{d_{u}+d_{v}}\\ =222\times \frac{1\times 2}{1+2}+2077\times \frac{1\times 3}{1+3}+911\times \frac{2\times 2}{2+2}+3440\times \frac{2\times 3}{2+3}+1233\times \frac{3\times 3}{3+3}\\ \approx 8594.25 \end{array}$$

$$\begin{array}{l} SC\left(G\right)=\sum_{uv\in E\left(G\right)}\frac{1}{\sqrt{d_{u}+d_{v}}}\\ =222\times \frac{1}{\sqrt{1+2}}+2077\times \frac{1}{\sqrt{1+3}}+911\times \frac{1}{\sqrt{2+2}}+3440\times \frac{1}{\sqrt{2+3}}+1233\times \frac{1}{\sqrt{3+3}}\\ \approx 3663.96 \end{array}$$

$$\begin{array}{l} So\left(G\right)=\sum_{uv\in E\left(G\right)}\sqrt{d_{u}^{2}+d_{v}^{2}}\\ =222\times \sqrt{1^{2}+2^{2}}+2077\times \sqrt{1^{2}+3^{2}}+911\times \sqrt{2^{2}+2^{2}}+3440\times \sqrt{2^{2}+3^{2}}+1233\times \sqrt{3^{2}+3^{2}}\\ \approx 27275.43 \end{array}$$

The degree-based topological indices for all fifteen structures were computed using a process analogous to the above, implemented in MATLAB, and are reported in Section 3.2. The fifteen degree-based topological indices computed for all fifteen antibody–antigen complexes are reported in Table 5. The index values vary substantially across the dataset, with 1S78 consistently producing the highest values and 4POU the lowest, across all fifteen indices.

**TABLE 5:** Topological indices for the 15 antibody–antigen structures.

Structure	$M_{1}$	$M_{2}$	$RM_{2}$	R	RR	HM	AZ	H	F	SD	ABC	GA	IS	SC	So
1S78	37216.00	42056.00	12723.00	3627.01	17858.67	180194.00	57638.52	3429.00	96082.00	19219.67	5751.46	7522.54	8594.25	3663.96	27275.43
2DD8	22996.00	26097.00	7964.00	2234.26	11044.73	111624.00	35858.98	2114.20	59430.00	11816.33	3542.76	4644.70	5319.60	2259.34	16841.34
2FJG	19434.00	22017.00	6701.00	1895.68	9335.34	94162.00	30370.27	1794.50	50128.00	9995.33	2999.18	3934.09	4496.83	1915.35	14231.14
3MJ9	24096.00	27252.00	8261.00	2351.89	11562.97	116740.00	37412.94	2223.17	62236.00	12450.67	3723.75	4871.21	5564.67	2373.83	17659.47
3U7Y	28800.00	32576.00	9883.00	2813.81	13828.88	139418.00	44894.75	2662.37	74266.00	14848.67	4450.79	5831.79	6658.93	2841.19	21096.44
4FP8	26436.00	29909.00	9074.00	2579.90	12689.11	128074.00	41111.69	2439.63	68256.00	13642.67	4083.71	5346.12	6107.98	2604.65	19370.62
4M5Z	23924.00	27158.00	8295.00	2324.60	11495.64	116086.00	37403.19	2201.23	61770.00	12269.33	3684.55	4836.50	5539.03	2351.79	17514.72
4POU	8738.00	9823.00	2945.00	860.13	4192.48	42110.00	13557.89	813.37	22464.00	4534.00	1357.34	1774.97	2017.27	866.75	6404.87
3WD5	21406.00	24203.00	7329.00	2086.11	10269.45	103730.00	33138.92	1971.30	55324.00	11064.67	3307.08	4323.23	4940.90	2106.27	15691.50
4ETQ	24874.00	28197.00	8588.00	2420.46	11948.10	120606.00	38814.72	2291.17	64212.00	12779.67	3834.41	5029.67	5755.08	2447.23	18215.59
4Y7M	13626.00	15396.00	4653.00	1324.71	6535.80	66012.00	21006.33	1251.60	35220.00	7044.00	2105.27	2749.79	3144.05	1338.85	9989.81
5C7X	19640.00	22247.00	6767.00	1914.55	9431.56	95186.00	30624.23	1811.57	50692.00	10112.33	3031.37	3972.83	4542.03	1934.32	14385.19
5CBA	11212.00	12679.00	3846.00	1097.31	5383.82	54260.00	17503.81	1038.30	28902.00	5783.33	1733.46	2271.84	2592.48	1107.34	8212.82
5GRJ	16342.00	18436.00	5559.00	1598.46	7836.11	79082.00	25252.77	1509.60	42210.00	8477.33	2530.15	3303.60	3768.38	1611.64	11984.26
5HGG	13920.00	15698.00	4736.00	1364.50	6683.97	67204.00	21680.95	1291.50	35808.00	7187.00	2156.22	2825.14	3218.42	1377.32	10196.79

### 3.3 Regression analysis against $pK_{d}$

**FIGURE 3:**
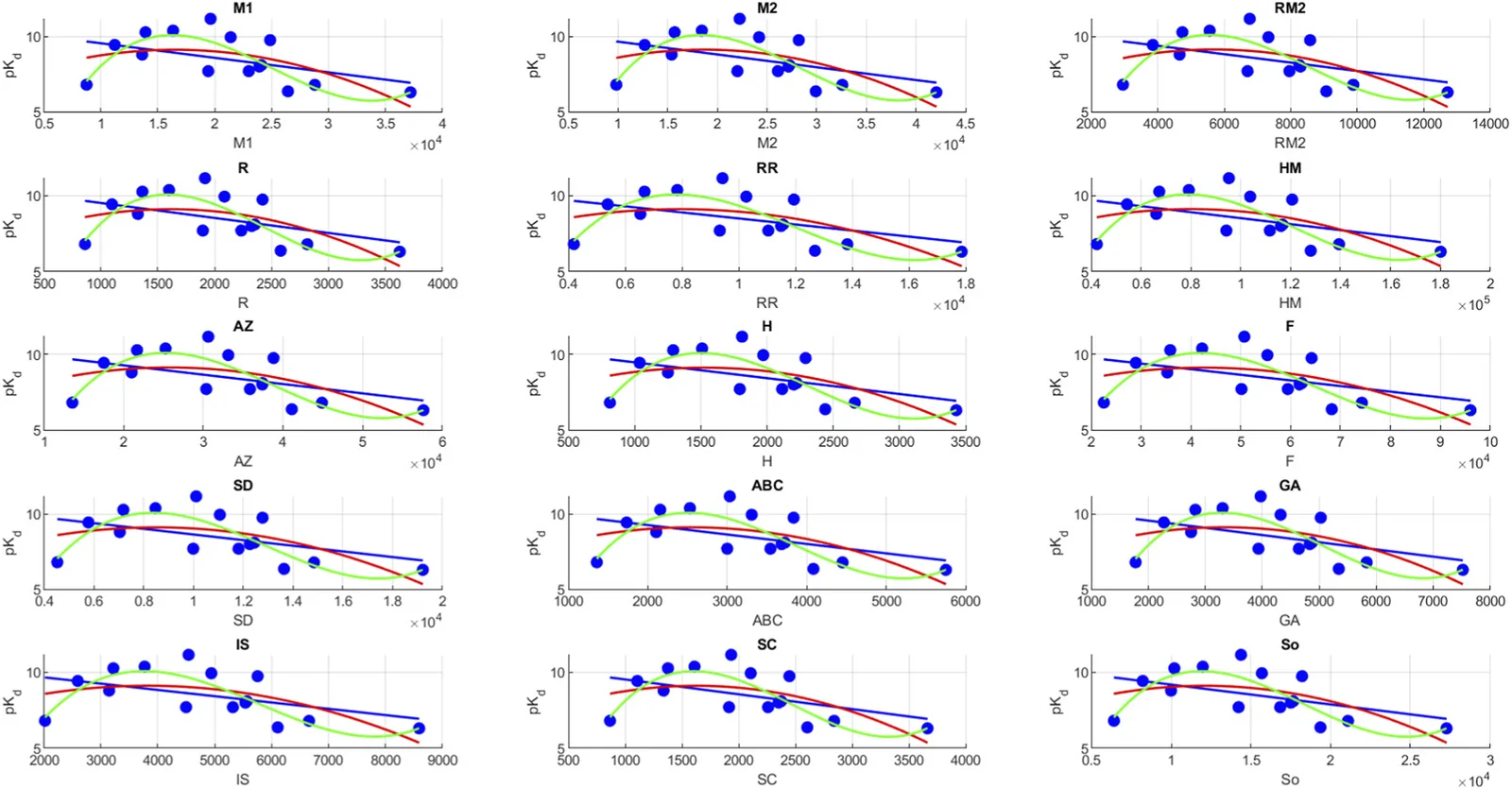
Regression plots against $pK_{d}$ values.

Regression analysis used $pK_{d}=-\log_{10}\left(K_{d}\right)$ as the main response variable for all fifteen degree-based topological indices. The results of the linear, quadratic, and cubic regression models are shown in Table 6, with the corresponding regression plots in Figure 3.

**TABLE 6:** Regression fit statistics for each model index and fit type versus ${\text{pK}}_{d}$ .

Index	Model	R	$R^{2}$	RMSE	p -value
$M_{1}$	Linear	0.4480	0.2007	1.3681	0.0940
	Quadratic	0.5939	0.3527	1.2311	0.0735
	Cubic	0.7796	0.6078	0.9584	0.0134
$M_{2}$	Linear	0.4473	0.2001	1.3687	0.0946
	Quadratic	0.5954	0.3545	1.2295	0.0724
	Cubic	0.7802	0.6087	0.9573	0.0132
$RM_{2}$	Linear	0.4460	0.1989	1.3697	0.0957
	Quadratic	0.5987	0.3584	1.2257	0.0697
	Cubic	0.7814	0.6106	0.9549	0.0129
R	Linear	0.4492	0.2018	1.3672	0.0930
	Quadratic	0.5944	0.3533	1.2307	0.0732
	Cubic	0.7805	0.6091	0.9567	0.0132
RR	Linear	0.4480	0.2007	1.3681	0.0940
	Quadratic	0.5943	0.3532	1.2307	0.0732
	Cubic	0.7798	0.6081	0.9579	0.0133
HM	Linear	0.4473	0.2001	1.3686	0.0946
	Quadratic	0.5946	0.3535	1.2304	0.0730
	Cubic	0.7797	0.6080	0.9582	0.0134
AZ	Linear	0.4481	0.2008	1.3681	0.0939
	Quadratic	0.5980	0.3576	1.2265	0.0703
	Cubic	0.7821	0.6117	0.9536	0.0127
H	Linear	0.4493	0.2018	1.3672	0.0930
	Quadratic	0.5948	0.3537	1.2302	0.0729
	Cubic	0.7807	0.6095	0.9563	0.0131
F	Linear	0.4474	0.2002	1.3686	0.0945
	Quadratic	0.5939	0.3527	1.2312	0.0735
	Cubic	0.7794	0.6074	0.9588	0.0135
SD	Linear	0.4486	0.2013	1.3676	0.0935
	Quadratic	0.5924	0.3510	1.2328	0.0747
	Cubic	0.7791	0.6069	0.9594	0.0135
ABC	Linear	0.4487	0.2014	1.3675	0.0934
	Quadratic	0.5932	0.3519	1.2319	0.0741
	Cubic	0.7795	0.6077	0.9585	0.0134
GA	Linear	0.4487	0.2013	1.3676	0.0934
	Quadratic	0.5943	0.3532	1.2307	0.0732
	Cubic	0.7801	0.6086	0.9574	0.0133
IS	Linear	0.4480	0.2007	1.3681	0.0940
	Quadratic	0.5947	0.3536	1.2303	0.0729
	Cubic	0.7800	0.6084	0.9576	0.0133
SC	Linear	0.4490	0.2016	1.3674	0.0932
	Quadratic	0.5942	0.3531	1.2308	0.0733
	Cubic	0.7802	0.6088	0.9571	0.0132
So	Linear	0.4480	0.2007	1.3681	0.0940
	Quadratic	0.5936	0.3524	1.2315	0.0738
	Cubic	0.7794	0.6075	0.9587	0.0134

For all fifteen indices, linear models produced $R^{2}$ values between approximately 0.198 and 0.201. None of the linear or quadratic models reached statistical significance at the p<0.05 level. Cubic models showed higher $R^{2}$ values, ranging from approximately 0.607–0.611, and were statistically significant for all indices. Notably, all fifteen indices performed similarly across every polynomial order. Despite their different mathematical forms, the indices produced $R^{2}$ values that stayed within about 0.01 across each regression type. This grouping is discussed further in Section 3.6.

### 3.4 Regression analysis against ΔG

Regression analysis was performed using the experimental Gibbs free energy of binding ΔG (kcal/mol) as an independent primary response variable. The ΔG values for the fifteen complexes are provided in Table 1, ranging from −8.45 kcal/mol (1S78) to −15.22 kcal/mol (5C7X). Since $\Delta G=RT\ln K_{d}$ , this variable is thermodynamically equal to $pK_{d}$ on a different scale. It offers an independent verification of the regression results in Section 3.3, using an experimentally derived value instead of a calculated one.

**FIGURE 4:**
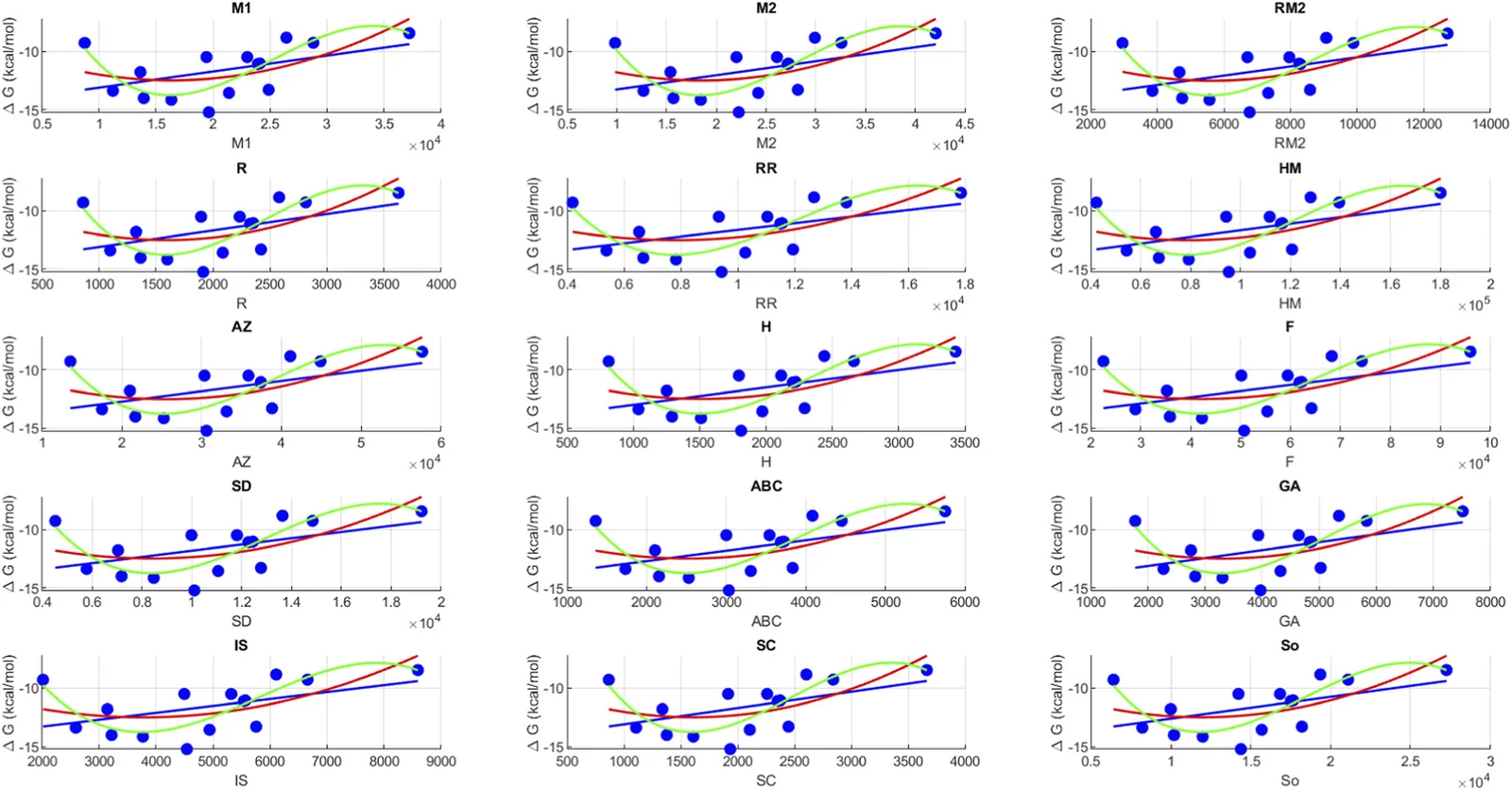
Regression plots against ΔG values.

The results for the linear, quadratic, and cubic regression models against ΔG are in Table 7, with the corresponding plots in Figure 4. Linear models produced $R^{2}$ values of approximately 0.21, while neither the linear nor the quadratic models achieved statistical significance. Cubic models produced $R^{2}$ values of approximately 0.597–0.602 and reached statistical significance for all fifteen indices. The close clustering of index performance across fifteen structurally distinct descriptors also appears in the ΔG analysis, further confirming the pattern seen with $pK_{d}$ .

**TABLE 7:** Regression fit statistics for each model index and fit type versus ΔG .

Index	Model	R	$R^{2}$	RMSE	p -value
$M_{1}$	Linear	0.4634	0.2147	1.8685	0.0819
	Quadratic	0.6063	0.3676	1.6767	0.0639
	Cubic	0.7731	0.5977	1.3374	0.0153
$M_{2}$	Linear	0.4625	0.2139	1.8694	0.0826
	Quadratic	0.6077	0.3693	1.6745	0.0629
	Cubic	0.7738	0.5987	1.3357	0.0151
$RM_{2}$	Linear	0.4609	0.2124	1.8712	0.0838
	Quadratic	0.6109	0.3732	1.6693	0.0606
	Cubic	0.7753	0.6011	1.3316	0.0146
R	Linear	0.4645	0.2158	1.8672	0.0811
	Quadratic	0.6068	0.3682	1.6759	0.0636
	Cubic	0.7740	0.5990	1.3352	0.0150
RR	Linear	0.4633	0.2147	1.8685	0.0820
	Quadratic	0.6067	0.3681	1.6761	0.0637
	Cubic	0.7733	0.5980	1.3368	0.0152
HM	Linear	0.4626	0.2140	1.8693	0.0825
	Quadratic	0.6070	0.3684	1.6757	0.0635
	Cubic	0.7733	0.5980	1.3369	0.0153
AZ	Linear	0.4630	0.2144	1.8689	0.0822
	Quadratic	0.6103	0.3725	1.6703	0.0611
	Cubic	0.7759	0.6020	1.3303	0.0145
H	Linear	0.4645	0.2158	1.8672	0.0811
	Quadratic	0.6072	0.3687	1.6753	0.0633
	Cubic	0.7742	0.5994	1.3345	0.0150
F	Linear	0.4628	0.2141	1.8692	0.0824
	Quadratic	0.6063	0.3676	1.6767	0.0640
	Cubic	0.7729	0.5974	1.3379	0.0154
SD	Linear	0.4641	0.2154	1.8676	0.0814
	Quadratic	0.6050	0.3660	1.6789	0.0650
	Cubic	0.7724	0.5967	1.3391	0.0155
ABC	Linear	0.4641	0.2154	1.8676	0.0814
	Quadratic	0.6057	0.3669	1.6777	0.0644
	Cubic	0.7729	0.5974	1.3378	0.0154
GA	Linear	0.4640	0.2153	1.8678	0.0815
	Quadratic	0.6067	0.3681	1.6761	0.0637
	Cubic	0.7736	0.5984	1.3362	0.0152
IS	Linear	0.4632	0.2146	1.8686	0.0820
	Quadratic	0.6071	0.3685	1.6755	0.0634
	Cubic	0.7735	0.5984	1.3363	0.0152
SC	Linear	0.4643	0.2156	1.8675	0.0813
	Quadratic	0.6067	0.3680	1.6762	0.0637
	Cubic	0.7737	0.5986	1.3358	0.0151
So	Linear	0.4634	0.2147	1.8685	0.0819
	Quadratic	0.6060	0.3673	1.6772	0.0642
	Cubic	0.7729	0.5973	1.3379	0.0154

### 3.5 Regression analysis against $K_{d}$

To provide completeness and comparison with the primary analyses in Sections 3.3 and 3.4, we conducted regression using untransformed $K_{d}$ (nM) as the response variable for the four indices that showed the strongest relationships in the primary analyses: the Augmented zagreb index, Reduced Second Zagreb index, Randić index and the Harmonic index. The results are in Table 8. Only the four highest-performing indices were retained because $K_{d}$ is presented solely as a secondary comparison.

**TABLE 8:** Regression fit statistics for the four best topological indices versus $K_{d}$ .

Index	Model	R	$R^{2}$	RMSE	p -value
AZ	Linear	0.5894	0.3474	127.2437	0.0208
	Quadratic	0.8216	0.6751	89.7881	0.0012
	Cubic	0.8385	0.7031	85.8332	0.0031
$RM_{2}$	Linear	0.5852	0.3424	127.7296	0.0219
	Quadratic	0.8212	0.6744	89.8737	0.0012
	Cubic	0.8374	0.7012	86.0975	0.0032
Randić	Linear	0.5956	0.3547	126.5337	0.0192
	Quadratic	0.8223	0.6761	89.6444	0.0012
	Cubic	0.8410	0.7073	85.2159	0.0028
Harmonic	Linear	0.5950	0.3540	126.5998	0.0193
	Quadratic	0.8221	0.6758	89.6877	0.0012
	Cubic	0.8406	0.7066	85.3218	0.0029

For untransformed $K_{d}$ , linear models produced $R^{2}$ values of approximately 0.342–0.354, while cubic models yielded $R^{2}$ values of around 0.701–0.707. All models were statistically significant (p<0.05) . These values are notably higher than those obtained for $pK_{d}$ and ΔG , especially at the linear level. However, as discussed in Section 2.2, this improvement reflects the scale and range of untransformed $K_{d}$ rather than a stronger structural signal.

### 3.6 Size benchmark analysis

**FIGURE 5:**
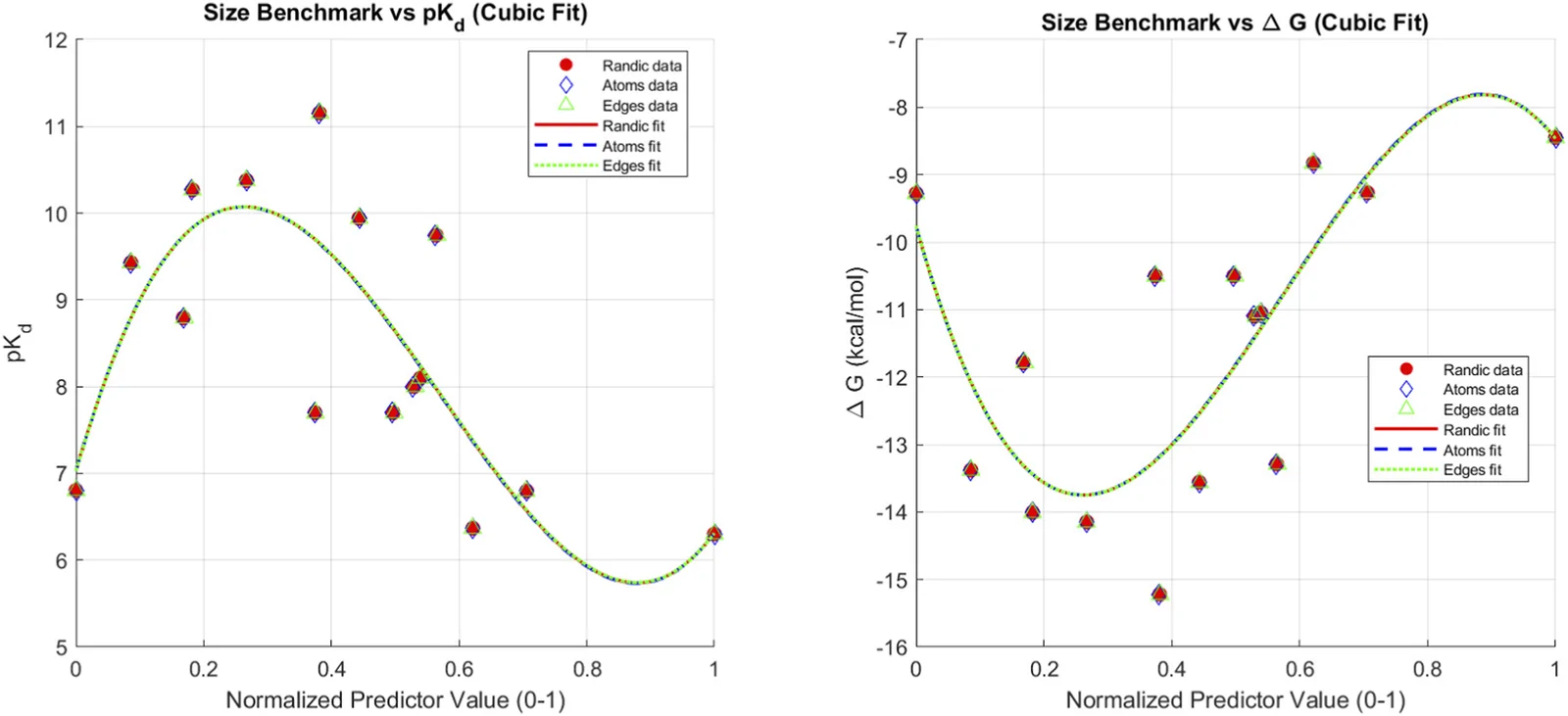
Representative overlay of a degree-based topological index (Randić), atom count and edge count.

To directly evaluate whether the associations seen in Sections 3.3 through 3.5 reflect information beyond overall molecular size, we extracted total atom count (N) and total edge count (E) from each preprocessed PDB structure using the graph construction method outlined in Section 2.3. We regressed these counts against $K_{d}$ , $pK_{d}$ , and ΔG using the same linear, quadratic, and cubic models. The values of N and E for all fifteen complexes are in Table 3, and the regression results given in Table 9. Figure 5 illustrates the regression curves for N , E , and the representative topological index, Randić index in relation to both $pK_{d}$ and ΔG . Since all fifteen indices exhibit nearly identical regression performance (Sections 3.3–3.4), the Randić index was selected as a representative example for graphical comparison. Table 9 gives the regression comparision. The similar performance of a simple size measure and the Randić index, consistent across $K_{d}$ , $pK_{d}$ , and ΔG , confirms that the associations in this dataset are due to molecular size rather than specific structural information represented by the indices. The same conclusion is reached regardless of whether the representative topological index is the classical Randić index or the marginally better-performing Augmented Zagreb index, as the performance differences among all fifteen descriptors are negligible.

**TABLE 9:** Regression fit statistics for size-based predictors (Atoms, Edges) versus $K_{d}$ , ${\text{pK}}_{d}$ , and ΔG .

Predictor	Response	Model	R	$R^{2}$	RMSE	p -value
Atoms (N)	$K_{d}$	Linear	0.5962	0.3555	126.4568	0.0190
	​	Quadratic	0.8224	0.6764	89.6100	0.0011
	​	Cubic	0.8415	0.7081	85.1088	0.0028
	$pK_{d}$	Linear	0.4492	0.2018	1.3672	0.0930
	​	Quadratic	0.5939	0.3527	1.2312	0.0736
	​	Cubic	0.7802	0.6088	0.9572	0.0132
	ΔG	Linear	0.4646	0.2158	1.8672	0.0811
	​	Quadratic	0.6063	0.3676	1.6767	0.0639
	​	Cubic	0.7737	0.5986	1.3358	0.0151
Edges (E)	$K_{d}$	Linear	0.5943	0.3532	126.6828	0.0195
	​	Quadratic	0.8216	0.6750	89.7928	0.0012
	​	Cubic	0.8402	0.7059	85.4238	0.0029
	$pK_{d}$	Linear	0.4487	0.2013	1.3676	0.0934
	​	Quadratic	0.5939	0.3527	1.2311	0.0735
	​	Cubic	0.7799	0.6083	0.9578	0.0133
	ΔG	Linear	0.4640	0.2153	1.8678	0.0815
	​	Quadratic	0.6064	0.3677	1.6766	0.0639
	​	Cubic	0.7734	0.5981	1.3367	0.0152
Randić (R)	$K_{d}$	Linear	0.5956	0.3547	126.5337	0.0192
	​	Quadratic	0.8223	0.6761	89.6444	0.0012
	​	Cubic	0.8410	0.7073	85.2159	0.0028
	$pK_{d}$	Linear	0.4492	0.2018	1.3672	0.0930
	​	Quadratic	0.5944	0.3533	1.2307	0.0732
	​	Cubic	0.7805	0.6091	0.9567	0.0132
	ΔG	Linear	0.4645	0.2158	1.8672	0.0811
	​	Quadratic	0.6068	0.3682	1.6759	0.0636
	​	Cubic	0.7740	0.5990	1.3352	0.0150

## 4 Discussion

Degree-based topological indices play an important role in QSAR and QSPR studies of small-molecule drug systems. They effectively capture significant structural variation in a way that is computationally easier and mathematically interpretable. This study aimed to explore whether this usefulness applies to larger biomolecular assemblies. Specifically, it looked into whether the connectivity patterns of antibody-antigen Fab complexes, as represented by degree-based indices, convey relevant information about binding affinity. The findings provide a clear and consistent answer, independently confirmed by two thermodynamically grounded response variables and a direct size benchmark.

The main regression analyses, which examined $pK_{d}$ and ΔG , showed that linear and quadratic models were not significant for all fifteen indices. However, cubic models were statistically significant for all fifteen indices across both response variables. This might suggest that degree-based connectivity captures some affinity-related structural information when viewed in isolation. Yet, the size benchmark makes this interpretation questionable. Total atom count and total edge count measurements that reflect only the number of atoms and bonds present produced $R^{2}$ values and significance levels matching those of the representative topological index within rounding error, for every polynomial order and all three response variables. An index that performs the same as a basic atom count is effectively measuring the same property. Consequently, the cubic level associations discussed in Sections 3.3 and 3.4 do not indicate a specific signal related to connectivity, they result from the sensitivity of both topological indices and size measures to overall molecular size, compounded by a weak association between molecular size and affinity within this dataset.

The secondary analysis on untransformed $K_{d}$ values showed notably higher apparent correlations, with linear $R^{2}$ values around 0.35 and cubic $R^{2}$ values nearing 0.7 for the best-performing indices. These figures might suggest a moderate to strong structure-affinity relationship if considered alone. However, the explanation lies in the scale of the response variable. The $K_{d}$ values in this dataset range nearly five orders of magnitude, from 0.007 nM to 500 nM. Ordinary least-squares regression applied to this untransformed scale gives disproportionate weight to a few high $K_{d}$ complexes at the top end of the range. Because binding free energy scales logarithmically with $K_{d}$ , the scale amplifies any size trend present in the data, inflating apparent correlations without revealing an underlying structural relationship. The $pK_{d}$ and ΔG analyses, by putting affinity on a scale correlated with binding free energy, negate this amplification. The resulting drop in correlation is informative, it highlights where the original apparent signal comes from instead of just indicating its absence.

Several aspects of the results support this interpretation. The most revealing is the close clustering of performance across all fifteen topological indices. Although the fifteen degree-based topological indices are mathematically distinct, they yielded almost identical regression performance, with $R^{2}$ values differing by only about 0.01 across all regression models and response variables. This suggests that they encode largely the same structural information for the antibody–antigen complexes considered in this study. If these indices were capturing genuinely distinct structural characteristics of the molecular graphs, one would expect a meaningful spread in their predictive performance. The almost uniform behavior suggests that all fifteen indices are influenced by the same underlying measure, which the size benchmark indicates is overall graph size.

The structural reason behind this outcome becomes apparent when considering the scale of the analysis. Each molecular graph in this dataset represents the entire antibody-antigen Fab complex, including heavy chain, light chain, and antigen, with total atom counts from 1826 (4POU) to 7703 (1S78). The binding interface, where molecular recognition and affinity occur, involves a much smaller group of atoms, typically involving only a small fraction of the total graph at the contact surface between antibody and antigen, representing only two to eight percent of the total graph. Framework regions, constant domains, and structural scaffolding away from the paratope make up the remaining bulk of edges and dominate every degree-based index, regardless of its mathematical form. The interface signal, no matter its size, is effectively averaged out by the overall connectivity of the surrounding structure. Under these conditions, indices become proxies for complex size, which the benchmark confirms.

This analysis also explains why the results differ so noticeably from the performance of degree-based indices in small-molecule QSAR applications. In a drug molecule with twenty to fifty atoms, the Randić and Sombor indices capture distinct aspects of local connectivity, such as branching patterns and ring closures, because these features represent meaningful parts of the entire graph. In a protein complex containing several thousand atoms, the same indices operate over a graph large enough that local topological features, although chemically relevant in small molecules, get overshadowed by the sheer number of edges in the larger structure. The scale at which the descriptor is computed, rather than the descriptor itself, poses the key limitation here.

These findings should not be interpreted as a limitation of graph-theoretical approaches for modeling biomolecular binding affinity. Rather, they identify a specific limitation associated with applying global degree-based topological indices to whole-complex molecular graphs. The benchmark analysis demonstrates that the structural information captured by these indices is largely explained by overall molecular size, rather than by connectivity patterns beyond size. This is an important finding because it clarifies the source of the limitation. Instead of suggesting that degree-based topological indices are inherently unsuitable for protein complexes, the results indicate that the primary issue lies in the scale at which the graph descriptors are computed. Consequently, future studies should focus on graph representations that emphasize local structural features, such as binding interfaces, or on size-normalized topological descriptors that better capture affinity-relevant structural information.

Several specific limitations of this study arise from this analysis and should be acknowledged. The dataset consists of only fifteen complexes, and with this small sample size, even cubic models using four parameters leave limited degrees of freedom for reliable conclusions. The size benchmark achieves the same statistical significance at the cubic level as the topological indices, meaning that the cubic-level significance noted in Sections 3.3 and 3.4 cannot be attributed to real connectivity information. However, a larger dataset could allow the same benchmark method to more effectively separate size effects from any genuine structural signal, should one exist after focusing on the interface. Furthermore, the molecular graphs in this study illustrate only covalent connectivity among the heavy atoms. Non-covalent interactions at the binding interface—like hydrogen bonds and van der Waals contacts—are not captured by the 1.9 Å threshold used here, yet these interactions are crucial for understanding affinity differences across complexes. A graph construction that focuses on interactions at the binding interface, using a larger distance threshold for atoms only at the contact surface, would simultaneously address both limitations at once.

Future research inspired by these findings has a clear direction. Limiting the graph to the atoms at the binding interface, defined, for example, by a 4–6 Å contact cutoff between antibody and antigen chain atoms, would eliminate the bulk dilution effect identified here and help the indices reflect connectivity patterns at the recognition site instead of averaged connectivity across the entire structure. Normalizing the indices by size such as expressing them per atom or per edge would also tackle the scale sensitivity shown by the benchmark. Both adjustments should be tested against $pK_{d}$ or ΔG , rather than untransformed $K_{d}$ , as this study clearly demonstrates why the latter is not suitable for this kind of analysis. An expanded dataset, drawing more broadly from the Pierce Laboratory benchmark, would allow for cross-validation and the application of model selection criteria like AIC or BIC. This could replace simple $R^{2}$ comparisons that are prone to overfitting with small sample sizes. Lastly, combining interface-restricted topological indices with geometrical descriptors of the binding surface—such as buried surface area, shape complementarity, or the number of interface hydrogen bonds—and physicochemical features like electrostatic complementarity within a multivariate regression or ensemble model might create a hybrid descriptor system. This approach would retain the interpretability and low cost of graph-theoretical methods while incorporating aspects of binding that connectivity alone cannot fully represent. Established predictors like AntiFormer, which integrates sequence information within a graph-based large language model framework, currently represent the best practice for predicting antibody-antigen affinity. The descriptors evaluated in this study are not suggested as alternatives to such models rather, the findings here clarify the conditions under which graph-theoretical structural features can add understandable and cost-effective components to hybrid frameworks that enhance sequence- and structure-based learning approaches.

## 5 Conclusion

Degree-based topological indices offer a simple and efficient way to describe molecular structure. This study explores their connection to antibody-antigen binding affinity using molecular graphs from fifteen experimentally determined Fab complex structures. After thorough preprocessing, which included altloc filtering, removing duplicated chains, and checking for valid vertex degrees, fifteen degree-based indices were calculated using an edge-partitioning method. These were compared against two main thermodynamic response variables, $pK_{d}$ and ΔG , along with untransformed $K_{d}$ as a secondary reference.

Linear and quadratic models were not significant across all fifteen indices when comparing them to $pK_{d}$ and ΔG . However, cubic models achieved statistical significance with $R^{2}$ values around 0.60. When comparing to untransformed Kd, much higher correlations were found, with cubic $R^{2}$ values nearing 0.71 for the best-performing indices. These results show that degree-based topological indices are indeed sensitive to structural differences in antibody-antigen complexes. They primarily reflect overall molecular size.

However, a size benchmark, where total atom count and total edge count were regressed against the same three response variables, demonstrated that these simple size measures matched the performance of the top-performing index closely, with only rounding errors across all polynomial orders and response variables. This outcome, consistent for $K_{d}$ , $pK_{d}$ , and ΔG , suggests that the relationships observed stem from overall molecular size rather than specific connectivity patterns at the binding interface.

The close clustering of results among all fifteen indices, despite their different mathematical approaches, supports the idea that whole-complex computation, rather than interface-restricted computation, is the main limiting factor. While this study does not rule out the potential of degree-based topological indices in modeling biomolecular affinity, it clearly outlines the conditions under which they might be effective: using interface-restricted graph construction, normalizing for size, regressing against suitable affinity measures, and combining with geometric and physicochemical descriptors of the binding surface in a hybrid modeling approach. This study sets the empirical foundation for more targeted and structure-informed investigations moving forward.

## Data Availability

The original contributions presented in the study are included in the article/supplementary material, further inquiries can be directed to the corresponding author.
